# Phylogenetic‐Pheromone Associations Obscured by Stabilising Selection and Natal Tree Effect in a Tree‐Killing Bark Beetle

**DOI:** 10.1111/mec.70226

**Published:** 2026-01-12

**Authors:** R. L. Isitt, J. A. Addison, S. B. Heard, D. S. Pureswaran

**Affiliations:** ^1^ Natural Resources Canada, Canadian Forest Service, Atlantic Forestry Centre Fredericton New Brunswick Canada; ^2^ Department of Biology University of New Brunswick Fredericton New Brunswick Canada

**Keywords:** host chemistry, pheromones, phylogeny, saltational shifts, stabilising selection

## Abstract

Insects are highly reliant on chemical cues such as pheromones to facilitate communication and navigation. Some of the roles of pheromones include attracting and finding mates and conspecifics, and in these cases, we expect stabilising selection to dampen within‐population pheromone variation. On the other hand, standing pheromone variation may lead to barriers to gene flow and saltational shifts that facilitate divergence and speciation. We investigated the relationships between pheromone variation and genetic variation in the spruce beetle, *Dendroctonus rufipennis* Kirby, a bark beetle that infests spruce. We found no convincing associations between genetic variation and pheromone variation in the spruce beetle. Instead, our results suggest that stabilising selection has acted to harmonise regional pheromone blends, including those of different sympatric clades, while pheromone blends differ regionally even within the same clade. Individual pheromone variation within regions cannot be attributed to phylogenetics and is instead partly explained by the identity of the natal tree, suggesting an environmental influence of host tree chemistry. Our results show that stabilising selection is not absolute, and that other opposing forces, such as co‐evolution and environmental influences, could contribute to within‐population variation.

## 1 Introduction

Among animals, insects are particularly reliant on chemical odours for communication and navigation (Gullan and Cranston 2010). It is therefore unsurprising that many insect species produce chemicals to facilitate such needs. In an intraspecific context, these chemicals or blends of chemicals are known as pheromones (Karlson and Lüscher 1959). Pheromones have varied roles, including mate finding, kin recognition, aggregation, enforcing social hierarchies in eusocial insects and alarm signalling (Yew and Chung 2015).

Because pheromones are often used to attract and find mates (sex pheromones) or conspecifics (aggregation pheromones), intraspecific variation in the production of or response to these types of pheromones can be mechanisms for assortative mating. As a result, variation in pheromone production among individuals is evolutionarily important and can even lead to reproductive isolation and eventual speciation (Wicker‐Thomas 2011). Insects produce pheromones either by enzymatic modification of endogenous metabolic products (de novo biosynthesis) or by modification and/or release of chemicals obtained from the environment (Tittiger and Blomquist 2017). Genetic and environmental variation may impact any part of this process, leading to variation in pheromone production both within and between species. Pheromones often consist of blends of multiple chemicals, which increases the potential for variation. For example, the bark beetle genera *Dendroctonus* and *Ips* together contain more than 30 species known to produce pheromone blends for mate finding and/or aggregative feeding, and more than a dozen different pheromone components have been documented between them. Rather than each species exclusively producing a unique single‐component pheromone, most *Dendroctonus* and *Ips* species produce multipart blends that vary in the presence/absence of specific components and their relative ratios (Symonds and Elgar 2004). This complexity in pheromone production means that variation in pheromone profiles among individuals or populations is easily generated—and this variation could either drive or retard genetic diversification (Wicker‐Thomas 2011). Such variation in pheromones has been suggested as a factor in the reproductive isolation of different populations of the European corn borer moth, *Ostrinia nubialis* Hübner (Thomas et al. 2003), but this possibility has not been addressed in bark beetles.

Pheromone variation among populations might evolve through one of two modes: gradual change that leads to conserved phenotypes among closely related lineages, or rapid saltational shifts resulting in distinct phenotypes between closely related lineages. Evidence from studies of lepidopteran, bark beetle, and *Drosophila* sex pheromones suggests a saltational mode of evolution: even closely related species tend to have considerable differences in pheromone blends. Saltational evolution of pheromone variation seems to be facilitated by relatively small changes to genes that produce considerable changes to the enzymatic pathways responsible for pheromone synthesis. These pheromone variants can arise from either selection or neutral evolution and may lead to reproductive isolation (Symonds and Elgar 2008).

Within populations, there are evolutionary forces that might either favour or oppose pheromone variation. Selection on pheromone profiles might be disruptive where there is a strong fitness advantage to avoiding predators and parasitoids that ‘eavesdrop’ on volatiles produced by their prey (Symonds and Elgar 2008). On the other hand, where rapid and efficient mate finding or aggregation has higher fitness value, stabilising selection should erode within‐population pheromone variation (Wicker‐Thomas 2011). This effect may be weakened if some individuals are pre‐adapted to prefer a novel pheromone (as in corn borer moth; Roelofs et al. 2002), but it is unknown how widespread such preadaptation is, or how often novel preferences are common or strong enough to outweigh reduced attraction of individuals preferring ‘normal’ pheromone profiles. It is thus not obvious how often and under what circumstances stabilising selection on pheromone profiles should predominate over diversifying selection and thus result in tightly focused pheromone production at the population level. Within‐population pheromone blend variation has been observed for several species, including the noctuid moth *Heliothis virescens* F. (Groot et al. 2014), the spruce beetle *Dendroctonus rufipennis* Kirby (Isitt et al. 2020) and the southern pine beetle *Dendroctonus frontalis* Zimm. (Pureswaran et al. 2008). As a further complication, variation in pheromone production can also be a consequence of variation in the availability of host plant‐produced biosynthetic precursors (Tillman et al. 1999; Tittiger and Blomquist 2017), in which case selective explanations at the level of insect populations aren't necessary.

The spruce beetle, *Dendroctonus rufipennis* , shows considerable variation in pheromone blend production, both within and between populations (Isitt et al. 2020). Furthermore, Maroja et al. (2007) discovered clear population structure, including two broadly sympatric Northern clades and one largely allopatric Rocky Mountain clade, with as much as 3%–4% COI sequence divergence between these clades. Maroja et al. (2007) propose that the cause of this differentiation was isolation of populations into at least three refugia during the last glacial maximum. Although the Northern clades are likely interbreeding at random, Maroja et al. (2007) found evidence of barriers to gene flow between regional populations and between the Rocky Mountain and Northern clades. A possible cause of these barriers to gene flow may be assortative mating in response to differences in pheromone blend. To test this hypothesis, we quantified the pheromone blends and mitochondrial COI sequences of individual beetles and tested for associations between pheromone blend variation, phylogenetics and geography. Secondarily, we asked whether the patterns of phylogeny and pheromone blend variation mirrored the saltational mode of pheromone evolution seen among different species of insects, including bark beetles.

Our results show that different clades of spruce beetle within a region tend to produce similar pheromone blends, while beetles of the same clade but from geographically distant populations tend to produce dissimilar pheromone blends. The best predictor of pheromone variation was the identity of the natal tree. Phenomena operating within local populations, such as stabilising selection, co‐evolution with conspecifics and predators and variable host chemistries, are the most convincing explanations for the pheromone variation in the spruce beetle. Conversely, pheromone variation is poorly correlated with genetic variation and is unlikely to cause considerable barriers to gene flow in the spruce beetle.

## 2 Materials and Methods

### 2.1 Study System

The spruce beetle ( *Dendroctonus rufipennis* Kirby) is a broadly distributed North American bark beetle that spends most of its life cycle within the phloem tissue of host spruce, where it feeds, reproduces and develops to maturity. Adult spruce beetles have an obligate winter diapause. When reared in the lab, adults need to be kept at or below 4°C for at least 70 days to complete this diapause and prompt a synchronised emergence upon rewarming (Bleiker and Meyers 2017; Bleiker and Willsey 2020). In nature, post‐diapause adults fly in the late spring or early summer in search of new hosts. Females are the ‘pioneering’ sex; they locate host trees, then attract conspecifics through the production of an aggregation pheromone. The known aggregation pheromone components of the spruce beetle are frontalin (1,5‐dimethyl‐6,8‐dioxabicyclo[3.2.1]octane; Dyer 1973, Gries et al. 1988), verbenene (4‐methylene‐6,6‐dimethylbicyclo[3.1.1]hept‐2‐ene; Gries et al. 1992), MCOL (1‐methyl‐2‐cyclohexen‐1‐ol; Borden et al. 1996) and seudenol (3‐methyl‐2‐cyclohexen‐1‐ol; Furniss et al. 1976, Vité et al. 1972). Western populations produce blends containing mostly frontalin, MCOL and verbenene, while eastern populations produce blends containing mostly MCOL and seudenol (Isitt et al. 2020). Evidence from trapping experiments suggests that this regional variation in pheromone production is matched by similar preferences in response (Pureswaran et al. 2024, unpublished data). Spruce beetles require feeding in a fresh host spruce before their hindguts contain detectable amounts of any pheromone component (Isitt et al. 2018).

### 2.2 Beetle Collection, Rearing and Feeding

In the spring of 2019, prior to beetle flight, collaborators felled five white spruce trees in each of three locations: Acadia Research Forest, NB (46.014 N, 66.344 W); Peace River, AB (56.622 N, 118.639 W); and Whitecourt, AB (54.008 N, 115.904 W). The Peace River and Whitecourt trees were left intact where they fell as “trap trees” to attract beetles. However, we suspected from prior years of efforts that beetle populations in Acadia Research Forest were very low. To improve the chances of attracting beetles in New Brunswick, we cut two bolts ~50 cm long from the lower bole of each of the trees and moved them into a small stand of spruce in Fundy National Park, NB (45.566 N, 64.984 W), where recent beetle attacks were observed on standing trees. Furthermore, we placed synthetic Atlantic spruce beetle lures (containing frontalin, seudenol and spruce terpenes; Synergy Semiochemicals Corp., Burnaby, BC, Canada) on wooden stakes approx. 5 m away from the bolts in Fundy National Park to attract spruce beetles to the bolts. The trap trees and bolts were successfully colonised by spruce beetles in all three sites.

We transported the infested bolts from Fundy National Park to the Atlantic Forestry Centre (AFC; Fredericton, NB) in early August 2019. The beetles were mostly late‐instar larvae. To expedite their development, we placed them into a greenhouse at AFC (6°C above ambient). By early November, the bolts from Fundy National Park contained adult beetles, at which point they were moved outdoors (covered by a tarp) to begin overwintering. The trap trees in both Alberta sites were left in the field until mid to late fall, after which three bolts were cut from the lower bole of each tree and shipped to the AFC. The bolts from Alberta contained spruce beetle larvae that were likely already in diapause, so we decided not to attempt to force a one‐year life cycle. We overwintered them outdoors, under a tarp, in the same manner as the bolts from Fundy National Park. COVID‐related delays prompted us to move all bolts (Alberta and New Brunswick) into a −4°C walk‐in freezer in late March 2020 to prevent uncontrolled outdoor emergence. Furthermore, the beetles in the Alberta bolts required a second season to develop into adults. After overwintering as larvae for approximately 9 months, we warmed them indoors (20°C–30°C) in early July 2020. The Alberta beetles had developed to adults by late August 2020, and we placed them back into the −4°C freezer for a final adult diapause.

We emerged beetles from the Fundy National Park bolts from August to October 2020. To do so, we warmed the bolts indoors (20°C–30°C) in groups of one to two trees, with individual bolts contained in plexiglass emergence cages. We collected newly emerged beetles daily, immediately separated them by sex, and briefly stored them in plastic petri dishes with moistened paper towel until used. We emerged beetles from the Whitecourt and Peace River (Alberta) sites from November 2020 to April 2021, following the same protocol.

To make the best use of limited numbers of beetles, we chose to use unpaired females only, which exhibit greater pheromone variation and complexity versus paired females or males (Isitt et al. 2018). For feeding the beetles, our collaborators cut six bolts from a single previously standing white spruce in each of the Whitecourt, Peace River and Acadia Research Forest sites in the fall of 2019. We stored these ‘feeding bolts’ in a freezer (−4°C) at AFC and thawed them individually as needed, 48 h before their use.

We typically placed newly emergent female spruce beetles into feeding bolts within 2 h of collection. To do so, we drilled a shallow ~2 cm tunnel into the phloem, nearly tangential to the curvature of the bolt, and gently inserted female beetles head‐first. After the beetles crawled the rest of the way into the tunnel, we plugged it with shredded phloem and stapled a piece of fabric mesh over the hole.

We allowed the beetles to feed for 18 h. We chose this duration to allow for a consistent daily schedule and because prior research suggested that feeding durations of 24 h or less may result in pheromone blends containing less MCH, thus being more likely to represent an aggregation pheromone blend (Isitt et al. 2018). After the beetles were fed, we extracted them by gently peeling back the bark of the feeding bolt. We placed the fed beetles individually into Cryotubes and chilled them in an ice bucket prior to pheromone extraction.

### 2.3 Pheromone Blend Quantification and Clustering

We extracted pheromones from fed beetles within an hour of their removal from the feeding bolts. We did this by pulling out the hindgut of each beetle with sharp forceps and placing it into a 2 mL GC–MS vial with a 200 μL glass insert containing 50 μL of a 4:1 mixture of pentane (≥ 99%, Fluka 76869) and hexane (≥ 99%, Fluka 52767), spiked with 5 ng/μL cycloheptanol (97%, Sigma‐Aldrich C98802) as an internal standard. We kept the vials at room temperature for 24 h to extract gut volatiles into the solvent, after which we stored them in a freezer (−20°C) until they were shipped off for chemical analyses. Prior to shipping, we removed the hindgut tissue from each sample by transferring the solvent into the outer GC–MS vial using a syringe, with the inserts discarded. We kept the remainder of each beetle frozen at −80°C until used for DNA extraction.

The Chemical Services Laboratory of the Pacific Forestry Centre (Victoria, BC) quantified the amounts of frontalin, MCOL, seudenol and verbenene in each sample using gas chromatography/mass spectrometry (GC–MS), matching retention times and the spectra of selected ions against analytical standards (purchased from Synergy Semiochemicals). Separation was done with a DB‐Wax chromatography column (30 m × 0.25 mm × 0.25 μm), with helium as a carrier gas (1.0 mL/min flow rate). The inlet was held at 200°C with a 5:1 split flow. The temperature programme was 40°C for 2 min, ramped to 130°C at 3°C/min, ramped to 240°C at 30°C/min, and held at 240°C for 5 min. Selected ion monitoring (SIM) ions were *m*/*z* 43, 72 and 100 for frontalin; 69 and 97 for MCOL; 84 and 97 for seudenol; and 91 and 92 for verbenene.

Following the methods in Isitt et al. (2020), we assigned each beetle a categorical pheromone profile using *K*‐means clustering of pheromone component ratios. Following clustering, we calculated *F*‐statistics (as per one‐way ANOVA) for each pheromone component between the clusters. *F*‐statistics are the ratios of between‐cluster variance to within‐cluster variance; we used their magnitudes to judge the relative ability of each pheromone component to discriminate between the clusters (Burns and Burns 2008).

### 2.4 DNA Extraction and Sequencing

We extracted DNA from individual spruce beetles and amplified a ~1.5 kb region of the COI gene following the methods in Maroja et al. (2007), with minor modifications. DNA was extracted from whole beetles (minus hindgut tissue) using the DNEasy Blood & Tissue Kit (QIAGEN Cat. No. 69506). We used primers TL2‐N‐3014 (5′‐TCCAATGCACTAATCTGCCATATTA‐3′) and CI‐J‐1718 (5′‐GGAGGATTTGGAAATTGATTAGTTCC‐3′) (Simon et al. 1994) for mtDNA COI purification. Polymerase chain reactions (25 μL) contained 2.5 μL of template DNA, 20 mM Tris HCl (pH 8.4), 50 mM KCl, 3 mM MgCl_2_, 0.2 mM dNTPs, 10 μM of each primer and 2.5 U of recombinant *Taq* DNA polymerase (Invitrogen). We used a Bio‐Rad C1000 Thermal Cycler with 35 cycles of 50 s at 95°C, 60 s at 48°C and 90 s at 72°C. The amplified DNA was sent to Genome Quebec (Montreal, Quebec) for Sanger sequencing (ABI 3730xl, Applied Biosystems). Primers TL2‐N‐3014 and CI‐J‐1718 were used for sequencing.

The sequences were aligned using the sangeranalyseR package (Chao et al. 2021) in R 4.3.2 (R Core Team 2023). They were aligned with and trimmed to the same length as the sequences provided by Maroja et al. (2007). Unique sequences have been deposited in GenBank (accession numbers PV661197–PV661486).

### 2.5 Phylogeny Reconstruction and Clade Assignment

We used IQ‐TREE 3.0.0 (Wong et al. 2025) with automatic model selection (ModelFinder; Kalyaanamoorthy et al. 2017) and maximum likelihood (ML) estimation to create the phylogenetic tree and genetic distance matrix from unique haplotypes. We used the Ultrafast bootstrap method (UFBoot; Hoang et al. 2018) with 5000 bootstrapped trees to estimate branch supports. We included both our own haplotypes, plus non‐duplicate *Dendroctonus rufipennis* haplotypes and the *D. murrayanae* outgroup from Maroja et al. (2007) (retrieved from GenBank) in the phylogeny reconstruction. To assign a clade to each haplotype, we used complete‐linkage clustering of the distance matrix, and extracted three clusters corresponding to the Northern I, Northern II, Rocky Mountain clades (Maroja et al. 2007), plus a fourth cluster for the outgroup.

### 2.6 Statistical Tests

We used multinomial logistic regression to determine if the pheromone profile produced by individual beetles could be predicted by clade membership, population and natal tree, with the natal tree factor being nested within populations. We used model selection between full and reduced models, including a null (intercept‐only) model, to determine whether there was support for each of the predictor variables in the multinomial logistic regression.

We followed up with multiple Fisher's exact tests to determine if pheromone profile assemblages were significantly different between clades within populations (three tests; one per population), between different populations within clades (two tests; one for the Rocky Mountain clade and one for the Northern I clade), and between different natal trees within populations (three tests; one per population). We adjusted *p*‐values for multiple comparisons within each set of tests using the Holm‐Bonferroni method.

To determine if there were significant correlations between pheromone production and population genetics at finer scales that may have been ignored by clustering techniques, we used a Mantel test between pheromone and genetic distance matrices. We used permutation tests with 9999 permutations to determine the significance level (*p*‐value) of the correlation and did so both with and without site‐wise stratification. This strategy allowed us to determine whether population genetic structure was confounded with geography, and to assess the significance of correlations between genetic and pheromone variation within sites. We expanded the maximum likelihood genetic distance matrix produced by IQ‐TREE from pairwise distances of unique haplotypes to pairwise distances of individual beetles. Because some haplotypes occurred in multiple beetles, this required duplication of rows and columns corresponding to these haplotypes. We assumed zero genetic distances between beetles with identical haplotypes. We populated the pheromone distance matrix using Euclidean distances of pheromone component ratios from individual beetles that produced a detectable amount of at least one pheromone component. Finally, we removed any beetles from the genetic distance matrix that did not occur in the pheromone distance matrix and identically arranged the rows and columns of both matrices. For the Mantel tests, we used Pearson correlations with 9999 permutations.

We used R 4.3.2 (R Core Team 2023) for all statistical tests, using the nnet library (Venables and Ripley 2002) for multinomial logistic regression and the vegan library (Oksanen et al. 2024) for Mantel tests.

## 3 Results

**FIGURE 1:**
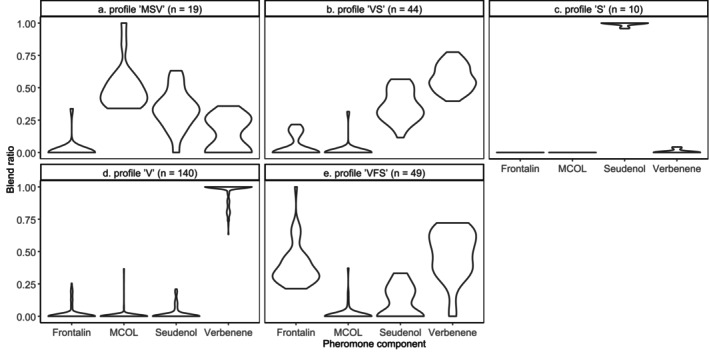
Blend ratios of aggregation pheromone components produced by 262 female spruce beetles, split into five clusters (pheromone profiles) by *k*‐means clustering. Pheromone profiles were named by concatenating the first letter of each non‐zero (by median) pheromone component in order of decreasing median.

Of the 290 female spruce beetles we sampled, 262 (29 from Fundy National Park, 58 from Peace River and 175 from Whitecourt) produced a detectable amount of one or more pheromone components. For *k*‐means clustering of pheromone blends (Figure 1), *k* = 5 was chosen based on the diminished reduction of within‐cluster sums of squares at higher values of *k*. This captured 84.2% of pheromone blend variation (between‐cluster sum of squares/total sum of squares). The *F*‐statistics (ratios of between‐cluster to within‐cluster variation) were 438 for verbenene, 384 for seudenol, 257 for MCOL and 182 for frontalin.

Our 290 spruce beetles provided 124 unique mtCOI haplotypes, 1114 bp in length. There were an additional 59 unique spruce beetle haplotypes and one outgroup haplotype ( *D. murrayanae* ) from Maroja et al. (2007). Among these 184 haplotypes were 242 variable sites, 137 of which were parsimony‐informative. There were no stop codons and no evidence of insertions or deletions. Automatic model selection identified TIM + I + R2 as the most parsimonious model: a transition model with unequal base frequencies, invariant sites and a two‐category FreeRate model of rate heterogeneity.

**FIGURE 2:**
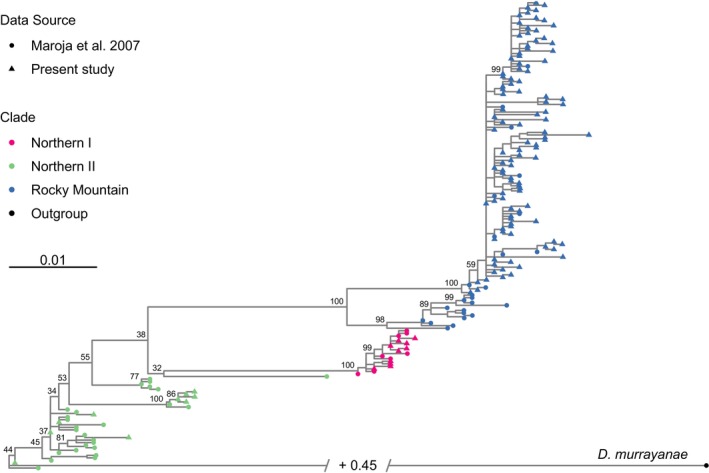
Maximum‐likelihood phylogeny of unique haplotypes of spruce beetle ( *Dendroctonus rufipennis* Kirby) from a 1114 bp region of mtCOI. Our study resulted in 124 unique haplotypes, with 60 additional haplotypes (including the *Dendroctonus murrayanae* Hopkins outgroup) obtained from Maroja et al. (2007). Note the break in the outgroup branch, which was shortened for clarity.

Spruce beetles collected from Whitecourt (AB), Peace River (AB) and Fundy National Park (NB) clustered into the same three clades as previously identified by Maroja et al. (2007). There was strong bootstrap support for monophyletic Northern I and Rocky Mountain clades. The Northern II clade was a basal paraphyletic clade in our maximum‐likelihood tree, but bootstrap support for this topology was low (Figure 2). This concurs with the consensus bootstrap tree in Maroja et al. (2007).

The Whitecourt and Peace River (AB) sites were dominated by beetles in the Rocky Mountain clade, with almost all beetles producing pheromone blends with verbenene as the largest component (profiles V, VS or VFS). Beetles from the Fundy National Park (NB) site were most associated with the Northern II clade and produced blends with a much greater proportion of MCOL and seudenol (represented by profiles MSV and S) than their western counterparts. A minority of beetles clustered into the Northern I clade in all three sites.

Model selection of multinomial logistic regressions suggests that the best model of pheromone profile depends solely on the identity of the natal tree, nested within geographic site (Table 1). The most parsimonious model that includes clade among the independent variables has lower accuracy than the best model and a ΔAICc of 23.

**TABLE 1:** Model selection of multinomial logistic regressions, with spruce beetle pheromone profile as the dependent variable and site, natal tree and genetic clade as independent variables. We based model selection on Akaike information criterion, corrected for small sample sizes (AICc). The quality of the model fit is given by the log‐likelihood (LL) and accuracy, the latter being the proportion of correct predictions made by the model. In the model column, a slash (/) indicates that the term on the right is nested within the term on the left.

Model	AICc	ΔAICc	LL	Accuracy
Site/tree	560	0	−201.97	0.65
Site	571	10	−272.75	0.59
Site + clade	583	23	−269.70	0.59
Site/tree + clade	583	23	−199.28	0.65
Site × clade	585	25	−265.97	0.60
Clade	603	43	−288.96	0.57
Null	670	110	−330.90	0.53
Site/tree × clade	691	131	−193.03	0.65

Beetles from the Rocky Mountain Clade did not exhibit significantly different pheromone profile assemblages between Peace River and Whitecourt, Alberta (Fisher's exact test: adjusted *p* = 0.097). In contrast, the pheromone profile assemblages of beetles from the Northern I clade were significantly different among the three sites (Fisher's exact test; adjusted *p* < 0.0001). There were no significant differences in pheromone profile assemblages for Fundy National Park (NB) beetles between the Northern I and Northern II clades (Fisher's exact test; adjusted *p* = 0.90), Peace River (AB) beetles between the Northern I and Rocky Mountain clades (Fisher's exact test; adjusted *p* = 0.65) or Whitecourt (AB) beetles between the Northern I and Rocky Mountain clades (Fisher's exact test; adjusted *p* = 0.66). There was a significant effect of natal tree on the pheromone profile assemblages produced by beetles in Whitecourt, Alberta (Fisher's exact test; adjusted *p* = 0.0015), but not in Peace River, Alberta (Fisher's exact test; adjusted *p* = 0.13) or Fundy National Park, New Brunswick (Fisher's exact test; adjusted *p* = 0.13). There was a significant correlation between genetic and pheromone distance matrices in an unstratified Mantel test (*r*_m_ = 0.30, *p* = 0.0001), but the significance of this correlation vanished when the permutation tests were stratified by site (*p* = 0.25).

## 4 Discussion

Pheromone blend variation in the spruce beetle appears to be under regional stabilising selection. Beetles from different clades in the same region produced similar pheromone blends despite significant variation between regions. For example, Northern I and II beetles from Fundy National Park produced blends dominated by MCOL and seudenol (profiles MSV and S), while Northern I and Rocky Mountain beetles from both sites in Alberta produced blends dominated by verbenene and frontalin (profiles V and VFS). In the case of the Northern I clade, these regional differences were particularly clear, with pheromone blends from Northern I beetles varying regionally and resembling those produced by their counterparts in other clades.

**FIGURE 3:**
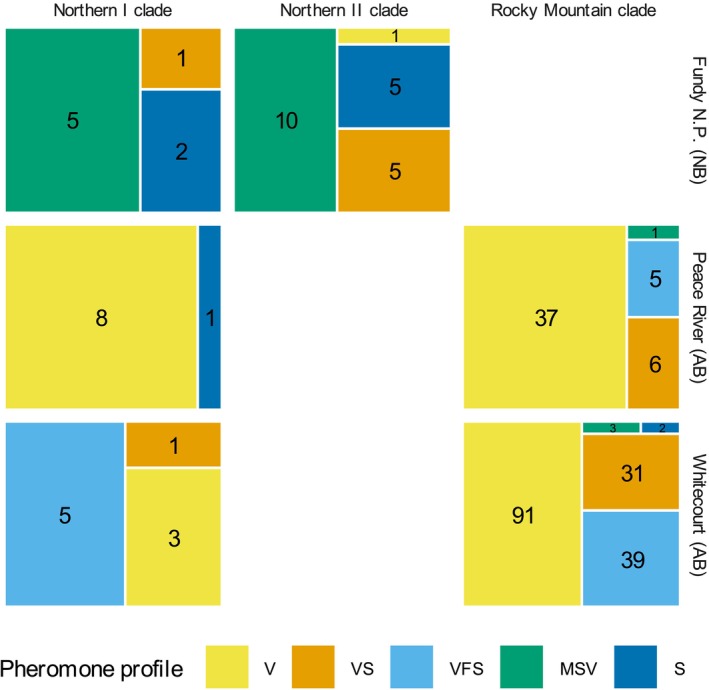
Assemblages of pheromone profiles from female spruce beetles ( *Dendroctonus rufipennis* ) split by genetic clade (based on mtDNA COI sequences) and geographic origin. Empty cells indicate that no beetles were collected in the given combinations of clade and origin. Numbers indicate the count of beetles that produced the indicated pheromone profile.

We interpret the *p*‐values of Fisher's exact tests with some caution. Fisher's exact tests may be overly conservative when group sizes are small, highly unbalanced, or when the experimental design violates the assumption of fixed row and column marginals, as ours did (Lydersen et al. 2009; Mehrotra et al. 2003). Consequently, we may have failed to detect true differences in pheromone profile frequencies between clades. The most obvious case where this may have occurred is between the Northern I and Rocky Mountain clades in Whitecourt, AB, which produced visibly different frequencies of the V and VFS pheromone profiles (Figure 3). Analyses of pheromone profiles also require caution, as the compositional differences between profiles are not equally dissimilar. The V and VFS profiles, for example, both share verbenene as the dominant pheromone component, while the V and S profiles are largely non‐overlapping.

To address some of the above concerns, we also conducted distance‐based analyses using a Mantel test, which measured the strength of correlations between genetic and pheromone distance matrices. This allowed us to preserve the variation within clades and pheromone profiles and to avoid splitting samples into unbalanced groups. When ignoring the geographical arrangement of samples, the Mantel test suggested a significant correlation between genetic and pheromone distance. However, we only found beetles from the Northern II and Rocky Mountain clades in New Brunswick and Alberta, respectively, confounding genetics with geography. When we stratified the Mantel test permutations by site, the correlation was no longer significant. This suggests a lack of association between population structure and pheromone blends within sites.

Lastly, as a more direct alternative to Fisher's exact tests, multinomial logistic regression allowed us to model the probability that a beetle produced a specific pheromone profile given its region, clade and natal tree ID. We found that the most parsimonious model of pheromone profile was based on the natal tree (nested within sites), while the best model that included clade as a predictor had a ΔAIC_c_ of 23, indicating a considerable lack of support (Burnham and Anderson 2004). Although knowing that a beetle comes from the Rocky Mountain clade allows a prediction of its pheromone blend, a more generalizable prediction can be made across multiple clades based on the site. This is the hallmark of stabilising selection, favouring intermediate phenotypes and decreasing pheromone diversity within a population.

Northern I was the only clade we documented in all three sites, and Maroja et al. (2007) show that it and Northern I and II are broadly distributed across North America. With Northern I having such a broad spatial distribution and clear regional differences in pheromone blends, we are struck by its lower genetic diversity (at most 0.9% within‐clade sequence divergence) compared to the Northern II and Rocky Mountain clades (3.8% and 4.2% sequence divergence, respectively). This contradicts what we would expect if pheromone blend variation were causing barriers to gene flow within the Northern I clade. Furthermore, all Northern I beetles emerged from natal bolts that were also occupied by Northern II or Rocky Mountain individuals. Maroja et al. (2007) found that the Northern I and Northern II clades were not significantly differentiated by their nuclear DNA. Thus, the Northern I clade may represent a retained ancestral mitochondrial lineage in an otherwise freely interbreeding Northern clade. In contrast, Maroja et al. (2007) found that the split between the Rocky Mountain and Northern clades was reflected in nuclear DNA. Whatever barriers to gene flow exist between the Northern and Rocky Mountain clades, our results suggest that neither (achiral) pheromone blend variation nor variation in microhabitat preferences are likely factors. We propose that differences in pheromone enantiomeric composition, courtship rituals or flight timing are plausible explanations worthy of further study.

Stabilising selection can explain why blends are similar within regions, but not why they differ between regions. Bark beetles often produce pheromone blends that contain the same or very similar components as produced by other species, in some cases with extremely similar qualitative blends (Symonds and Elgar 2004; Sullivan 2024). Attraction of multiple species to a single pheromone blend has been documented for many insect taxa, including stink bugs (Tillman et al. 2010), pine tip moths (Berisford et al. 1979) and bark beetles (Lu et al. 2007). The need for beetles to distinguish conspecifics from heterospecifics to avoid mate‐finding errors may drive regional differences in pheromone blends when two or more species would otherwise have similar blends. Another possible selective pressure is caused by the response of predatory species to the volatiles produced by their bark beetle prey. Over time, this could be expected to select for less conspicuous pheromone blends, or blends containing components that predators do not respond to. While this selective force should operate locally, predator communities vary geographically, and so too would these selective pressures. This is believed to be the case for *Ips pini* , whose geographic pheromone blend variation may be driven by co‐evolution with predators that find their prey via those same volatiles (Symonds and Elgar 2008). The standing variation in pheromone blends that we observed within spruce beetle populations would likely facilitate rapid pheromone evolution in response to changing heterospecific interactions (Barrett and Schluter 2008). Similar situations may underpin the saltational evolution of insect pheromones demonstrated by other authors (Symonds and Elgar 2008).

For selection to act on pheromone blends within populations, there must be a genetic basis for pheromone production. Whatever that genetic basis may be, we were unable to detect it from our MT‐COI lineages. The rate of mutation of MT‐COI is typically high enough to distinguish between species and to reveal population structure within species, but it is also known to be highly conserved between conspecifics (Hebert et al. 2003). Thus, a possible explanation for our results is that pheromone biosynthesis is driven by a relatively small number of genes and SNPs therein, which are more variable among conspecifics than MT‐COI. The high degree of pheromone variation that we observed among closely related beetles, including those with identical MT‐COI haplotypes, is at least consistent with this hypothesis.

However, we must also consider environmental influences on pheromone production. In our analyses, the spatial grouping of the natal tree outperformed all other factors in predicting pheromone blend variation. In both this study and in a prior study (Isitt et al. 2020), we saw significant pheromone profile variation between natal trees within the same site. These trees were harvested from the same stands, tens to hundreds of metres apart at most, so we expect that the trees were infested by beetles from the same population. Although we cannot be certain of the exact reason for this effect, we suspect the influence of host tree chemistry. The chemical environment of the host tree phloem varies regionally and locally from tree to tree (Pureswaran et al. 2004), and many bark beetle species produce pheromone components that are derived from host tree terpenoids. This likely includes the spruce beetle, which may derive verbenene from α‐pinene, though some evidence suggests de novo production (Tittiger and Blomquist 2017). Verbenene was not only the most commonly produced aggregation pheromone component by our beetles, it was also the pheromone component that best distinguished between the pheromone profiles (as per *F*‐statistics). Verbenene was clearly an influential component of pheromone variation in our study and its production may have been impacted by the α‐pinene availability of the beetles' natal trees.

Unfortunately, as our beetle collections were driven by opportunity (elevated populations/outbreaks of the spruce beetle), the geographic range of our sampling was limited to only three sites and two provinces. By comparing our sequence data to Maroja et al. (2007), we are reasonably confident that we captured a wide range of the genetic variation that exists for the species, assuming that the undersampled central region of the boreal forest is also dominated by the Northern I and II clades found in the east and west. However, prior work documents unique pheromone blends in beetles from BC, different regions of Alberta and different Atlantic provinces (Isitt et al. 2020), and so we expect that much additional pheromone variation remains undiscovered. We therefore hypothesise that regional pheromone variation is widespread across the species' range, while the broad‐scale population genetics are dominated by the Rocky Mountain versus Northern split.

Our results suggest that pheromone blend variation in the spruce beetle is driven by local selective pressures and stabilising selection that set up regional differences in pheromone blend variation, while reducing variation between different clades in sympatry. There is also considerable individual variation, including between closely related individuals, that is likely driven in part by the chemical environment of the host trees. Even host tree chemistry, however, *could* effectively be heritable among beetles if there is genetic variation among females in their host choice. Whatever the mechanisms, if some individual variation in pheromone blends has a genetic basis, it could facilitate rapid evolution of populations in response to changes in their chemical ecology, such as range shifts in populations of predators that locate their prey by ‘eavesdropping’ on the volatile chemicals they produce.

High individual variation in pheromone production may mirror similarly high individual variation in pheromone response; otherwise, we would expect individuals producing less common pheromone blends to be at a disadvantage for attracting mates and/or conspecifics (Wicker‐Thomas 2011). High individual variation in pheromone response may also facilitate introgression between populations despite regional differences in pheromone blend, similar to the broad male response in the fall armyworm that enables interbreeding despite different female‐produced pheromone blends (Sisay et al. 2024).

The tension between natal‐tree effects (which increase both local and regional pheromone variation) and stabilising selection (which constrains it, but only locally) provides an interesting example of the complexity of insect chemical ecology, and makes it difficult to predict evolutionary trajectories in these insects. Pheromone‐based aggregation and mate choice have the potential to either accelerate local and regional diversification or to retard it, and our work with *Dendroctonus* illustrates some of the factors that may influence such outcomes. The rich dynamics that are likely to result also complicate efforts to manage these ecologically and economically important insects. However, our results with *D. rufipennis* suggest a path forward, combining population genetics and chemical assays to discover the relationship between ancestry and environment in shaping the pheromones underlying aggregation and mate‐selection behaviour.

## Author Contributions

R.L.I., S.B.H., and D.S.P. conceived the project. J.A.A. provided lab space, equipment, and expertise for genomic techniques. R.L.I. performed the lab work and analyses, created visualizations, and wrote the first draft. All authors contributed to manuscript editing.

## Funding

This research was funded by an NSERC Discovery Grant RGPIN‐2018‐04065 and Canadian Forest Service Operating Funds to DSP and an NSERC Discovery Grant RGPIN‐2015‐04418 to SBH.

## Conflicts of Interest

The authors declare no conflicts of interest.

## Supporting information


**Data S1:** mec70226‐sup‐0001‐Supinfo.zip.

## Data Availability

A zip archive including data and scripts necessary to replicate these analyses are included as Supporting Information. Unique sequences were deposited in GenBank (accession numbers PV661197–PV661486).
